# Considerable variation in the 95-95-95 targets accomplishment between children and adults might delay achievement of set targets

**DOI:** 10.3389/fpubh.2025.1565242

**Published:** 2025-04-29

**Authors:** Benard W. Kulohoma, Colette S. A. Wesonga

**Affiliations:** ^1^IAVI Africa, Nairobi, Kenya; ^2^Ortholog, Nairobi, Kenya

**Keywords:** children, HIV, 95–95–95 HIV/AIDS goals, challenges, Africa

## Abstract

Despite a significant reduction in the global HIV disease prevalence in recent years, children under 15 years of age still account for 3% of people living with HIV, 9% of new incidence, and 12% of AIDS-related deaths. Although there is increased access and use of antiretroviral drugs, children under 1 year in resource-poor settings with a high HIV disease burden remain vulnerable due to poor initiation of these critical interventions impeding progress to meet the 95–95–95 targets. There are renewed efforts to ensure that exposed children under 15 years are not left behind by scaling diagnostics and clinical management in the most affected communities. However, gaps remain in the integration of these services into maternal, child, and adolescent healthcare services within these communities, resulting in only 67% of HIV-exposed infants being tested within 2 months of birth, globally in 2023. Consequently, only 29% of all exposed children under 15 years were initiated in antiretroviral treatment before their 5th birthday in 2023. There are successes for adults aged 15 years and above, but children under 15 years risk being left behind in achieving the 95–95–95 targets. In this study, we review efforts made to reduce these substantial regional variations when comparing progress made between children under 15 years and adults and highlight gaps that might impede achievement of the 95–95–95 targets among children.

## Introduction

We are at the midpoint toward the United Nations’ established goal of ending the AIDS epidemic by 2030. However, despite considerable progress in decreasing HIV incidence, challenges remain in accomplishing this goal. These challenges include diminishing funding for the HIV/AIDS response, stigma, discrimination, human rights violations, and constrained health systems, especially in resource-poor settings with a significant burden of HIV disease, that threaten success (1–3). Unlike other infectious diseases, for example malaria and TB, HIV cannot be eliminated without a cure or a vaccine, and vaccine research and development efforts have been impeded by the complex nature of the constantly evolving causative viral pathogen (4, 5). In the absence of a vaccine, antiretroviral drugs (ARVs) are used to treat HIV by reducing viral loads, decreasing the risk of disease transmission to near zero (6). This has improved longevity among people living with HIV, enabling a new span where discordant couples can have healthy children without HIV (7). However, not all of the 39.9 million people living with HIV worldwide are receiving this necessary treatment, with a disproportionate number of those not accessing treatment living in marginalized regions with high HIV disease burden (8, 9). ARV treatment programs are missing 9.3 million (23%) people who require this lifesaving treatment, with children (0–14 years) being the most affected (10). There is an urgent need to reduce HIV-related morbidity and mortality among this vulnerable population.

## The UNAIDS 95–95–95 strategy

**Figure 1:**
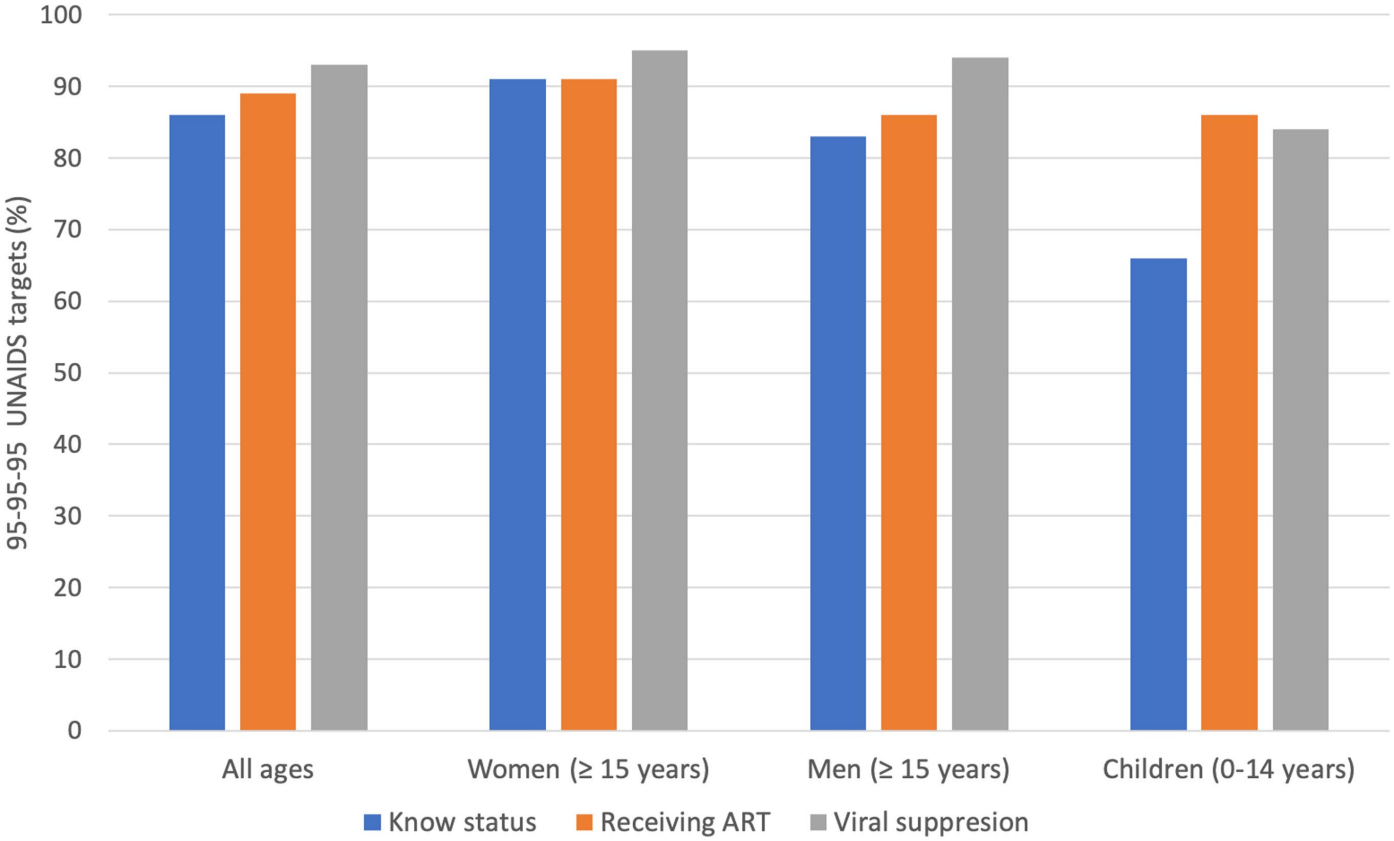
95–95–95 UNAIDS targets. The achievements were made across all ages, and there is disaggregation among women, men, and children. The goals aim for 95% of all people living with HIV to know their HIV status; among these people who know their status, 95% have sustained access to antiretroviral therapy (ART); and 95% of the people receiving ART achieve viral suppression.

One of the strategies to end the HIV epidemic entails achieving the 95–95–95 target scheme for testing and treatment. This ensures that 95% of all people living with HIV know their HIV status; among those who know their status, 95% have sustained access to antiretroviral therapy (ART); and 95% of those receiving ART achieve viral suppression (11). This has shifted focus to HIV treatment, making it more person-centered, and expanded access to treatment as an essential public health intervention (11). The current progress made against the target for all ages is 86–89–93, with 91–91–95 among women (15 years and above), 83–86–94 among men (15 years and above), and 66–86–84 among children (0–14 years), indicating that children risk being left behind (Figure 1) (8, 10, 12). Botswana, the country with the third highest HIV prevalence in the world, has exceeded the 95–95–95 targets, highlighting variations between national HIV responses, even in regions with a disproportionate disease burden (13, 14). There is regional variation in advancements toward the 2025 95–95–95 targets in children compared to adults, and only in South Asia (children: 94–93–77 vs. adults: 74–63–62) and Eastern Europe and Central Asia (children: 75–73–63 vs. adults: 60–51–43) is there more progress in children compared to adults (12).

## Challenges in reaching 95–95–95 targets in children (0–14 years)

Greater effort is required in many developing countries to achieve epidemic control among children. Inclusion of all demographics into HIV care, which also comprises children, and consideration of their testing, the treatment needs will not only help achieve the goal of ending the HIV epidemic as a public health threat but also have economic gains in the long term. Although some developing countries are making progress toward the 95–95–95 target, there might be children missing from these data due to loss of follow-up, especially among older children (>18 months old), underestimating the actual prevalence levels, which risks undermining the success accomplished (15).

The percentage of pregnant and breastfeeding women receiving antiretroviral therapy (84%) presently remains comparable to the levels in 2019 (10, 16, 17). ART among pregnant women is important for preventing perinatal and sexual HIV transmission. Globally, increased ART among pregnant women has resulted in a 50% reduction in perinatal infections, with infants acquiring perinatal HIV infection (48%, *n* = 76,800) born to mothers who are either not on ART due to lack of knowledge of their HIV status, acquired HIV during pregnancy or breastfeeding, interrupted treatment during pregnancy or breastfeeding, or did not achieve adequate viral suppression during ART (18). ART has prevented approximately 2.1 million child deaths since 2000; however, despite a marked decline in the number of new HIV infections, 120,000 new infections still occurred among children in 2023 due to vertical disease transmission (10, 16). Overall, the drop in the number of new HIV infections among children is driven by drops in eastern and southern Africa, which had 73% fewer infections in 2023 compared to 2010 (10). This contrasts findings in western and central Africa with modest (44%) declines, and these regions now account for 41% of the HIV incidence among children (10). Consequently, we are behind the target of viral suppression among 75% of children living with HIV, currently at only 48% globally (10). Poor knowledge of mother-to-child transmission of HIV, non-attendance at antenatal clinics, and inaccessible health facility delivery significantly contribute to vertical HIV transmission, and at least one HIV test is recommended for every woman during pregnancy (17).

Children (0–14 years) make up 3% of all people living with HIV, 9% of new incidences, and 12% of AIDS-related deaths (12). Children under 1 year in resource-poor settings with high HIV disease burden remain vulnerable due to poor initiation and access to treatment. In 2023, only 67% of HIV-exposed infants were tested within 2 months of birth, and consequently, only 29% of all exposed children under 15 years were initiated in antiretroviral treatment before their 5th birthday, with more older children entering ART programs (10, 12). WHO guidelines recommend the initiation of lifelong antiretroviral therapy (ART) in all children living with HIV below 5 years, notwithstanding their immune response, to improve access and disease outcome (19).

In 2023, only 48% of children living with HIV achieved viral suppression, and poor ART treatment adherence has been associated with loss of follow-up and continues to pose a great challenge, especially in developing countries (12, 20). It is, therefore, important to identify reasons for disengagement from HIV care services and remove barriers to enable re-engagement interventions tailored to the needs of children (21–26).

Children are less likely to have access to ART compared to adults, and closing this gap will ensure children living with HIV receive lifelong treatment to lead healthy lives (27). Poorer adherence to ART in children compared to adults, especially those in rural settings, can be improved by counseling targeting their parents and caregivers (27, 28). Non-adherence has been associated with poor ART outcomes that include drug resistance, treatment failure, and mortality in children and adolescents living with HIV (28–34). Moreover, children have fewer drug formulation options available and need to be considered during the development of new interventions to impede treatment failure (29, 35, 36). Limited drug formulation options make children particularly vulnerable to ART failure due to the emergence of drug-resistant HIV-1 quasispecies, which have the potential to compromise second-line therapy efficacy (37, 38). The genetic diversity of the constantly evolving HIV has been evaluated using molecular modeling approaches to understand subtype-specific drug interactions to provide insights into strategies that would optimize the limited pediatric ART regimens available. These findings show significant variation in drug-subtype binding, underscoring the importance of tailored treatment approaches, especially for children (39).

Unlike other infectious diseases, for example malaria and TB, HIV cannot be eliminated without a cure or a vaccine, and vaccine research and development efforts have been impeded by the complex nature of the constantly evolving causative viral pathogen (4, 5).

HIV and TB are both infectious diseases of pressing global health concern. In children, co-infection with TB and HIV may delay diagnosis due to atypical presentation in patients, and treatment requires consideration of potential TB and HIV drug-to-drug interactions and delays initiating ART during TB treatment, especially in children with advanced immunosuppression (40). Therefore, a cohesive approach is required when managing pediatric patients with comorbidity, especially in resource-constrained settings (41).

Discovery medicine often does not prioritize diseases affecting low- and middle-income countries (LMICs), and there are fewer product development pipelines and subsequent clinical trials inclined toward diseases in these countries. This poses a challenge to access to treatment products primarily because they are not registered or licensed in LMICs to expand product access to lifesaving treatments. An example is the growing concern over access to the pre-exposure prophylaxis (PrEP) injectable drug, lenacapavir, given every 6 months that has been shown to have 100% efficacy by vulnerable and marginalized populations in developing countries (42–44).

HIV vaccines remain important strategies that are still under development for use alongside ART to ensure an AIDS-free generation. However, children have largely been left out of HIV vaccine research, and inclusion in immune-based clinical trials is essential to evaluate safe vaccines providing perinatal protection administered passively to pregnant women or actively to infants at birth to prevent vertical HIV transmission (45, 46). The huge costs of vaccine manufacturing and weak or outmoded regulatory processes in developing countries, which are not optimized to support novel discovery medicine vaccines and treatment interventions critical for the protection of vulnerable children, also compound the hurdles to disease prevention (47).

There is now an emphasis on devolving pediatric diagnosis and clinical management of children exposed to or living with HIV (12, 48). This will enable integration with maternal and child health services at facilities to be more easily accessed by communities at the sub-national level, reducing barriers to the HIV response among children (11, 49–51).

Efforts to achieve the goal of ending the AIDS epidemic by 2030 require financial resources and investments on a global scale. In 2023, there was a $358 million decrease in donor government funding compared to a total of $8.22 billion available in 2022 (52). A total of $19.8 billion was available for the AIDS response in low- and middle-income countries against $29.3 billion required annually to end the epidemic by 2030 (8, 10). Targeted interventions and further investment are required to reach the 95–95–95 targets, without which the AIDS response will be reversed, and the current progress will diminish as incidence and mortality begin to overtake the response (50). At present, the HIV epidemic response requires continued and increased investment to ensure no child has AIDS (53).

Armed conflict is a unique challenge that ravages healthcare infrastructure and systems, leading to an increased HIV incidence and prevalence (54). A sustained HIV response, difficult as it is, is necessary in conflict and post-conflict to ensure preventing new infections. An example is the end of the civil war in Liberia, which resulted in a decline in HIV prevalence among children and women attributed to the establishment of a National AIDS Control Program. The success in Liberia can be attributed to the program that provided counseling and voluntary testing, prevention of mother-to-child transmission, and an expanded ART program (55).

## Conclusion

In conclusion, addressing the unique challenges faced by children living with HIV is essential to ensure they are not left behind in achieving the 95–95–95 target. Improved access to life-saving ART, adherence support during treatment, inclusion in new intervention and vaccine development, better integration of pediatric HIV diagnosis and clinical management, and continuous resource mobilization are some of the concerted efforts that are important for bridging the gap between children and adults. This will enable the realization of a future where children living with HIV can lead long, healthy lives, ending AIDS as a public health threat.
